# A comprehensive analysis of the WRKY family in soybean and functional analysis of GmWRKY164-*GmGSL7c* in resistance to *soybean mosaic virus*

**DOI:** 10.1186/s12864-024-10523-8

**Published:** 2024-06-19

**Authors:** Zhihua Zhao, Rongna Wang, Weihua Su, Tianjie Sun, Mengnan Qi, Xueyan Zhang, Fengju Wei, Zhouliang Yu, Fuming Xiao, Long Yan, Chunyan Yang, Jie Zhang, Dongmei Wang

**Affiliations:** 1 State Key Laboratory of North China Crop Improvement and Regulation/Hebei Key Laboratory of Plant Physiology and Molecular Pathology, College of Life Sciences, Hebei Agricultural University, Baoding, 071001 China; 2 School of Life Sciences, Yunnan University, Kunming, 650500 China; 3 Handan Municipal Academy of Agricultural Sciences, Hebei Province, Handan, 056001 China; 4 Institute of Cereal and Oil Crops, Hebei Academy of Agriculture and Forestry Sciences, Shijiazhuang, 050031 China

**Keywords:** Soybean, SMV, WRKY gene family, Callose deposition

## Abstract

**Background:**

Soybean mosaic disease caused by *soybean mosaic virus* (SMV) is one of the most devastating and widespread diseases in soybean producing areas worldwide. The WRKY transcription factors (TFs) are widely involved in plant development and stress responses. However, the roles of the GmWRKY TFs in resistance to SMV are largely unclear.

**Results:**

Here, 185 *GmWRKYs* were characterized in soybean (*Glycine max*), among which 60 *GmWRKY* genes were differentially expressed during SMV infection according to the transcriptome data. The transcriptome data and RT-qPCR results showed that the expression of *GmWRKY164* decreased after imidazole treatment and had higher expression levels in the incompatible combination between soybean cultivar variety Jidou 7 and SMV strain N3. Remarkably, the silencing of *GmWRKY164* reduced callose deposition and enhanced virus spread during SMV infection. In addition, the transcript levels of the *GmGSL7c* were dramatically lower upon the silencing of *GmWRKY164*. Furthermore, EMSA and ChIP-qPCR revealed that GmWRKY164 can directly bind to the promoter of *GmGSL7c*, which contains the W-box element.

**Conclusion:**

Our findings suggest that GmWRKY164 plays a positive role in resistance to SMV infection by regulating the expression of *GmGSL7c*, resulting in the deposition of callose and the inhibition of viral movement, which provides guidance for future studies in understanding virus-resistance mechanisms in soybean.

**Supplementary Information:**

The online version contains supplementary material available at 10.1186/s12864-024-10523-8.

## Background

Soybean mosaic disease caused by SMV is one of the most common viral diseases worldwide. SMV can cause severe soybean yield decline and seed quality deterioration [1]. Although several SMV strains and quantitative trait loci (QTLs) related to SMV resistance in soybean have been identified, mechanistic studies on the function and regulation of the antiviral genes are rare [2, 3]. Plasmodesmata (PDs) are plasma membrane-lined channels that connect adjacent cells to mediate symplastic communication in plants [4]. Callose, a beta-1,3 glucan that can be reversibly deposited around the neck of PD, modifies cell wall properties and restricts PD aperture that affects the transportation of signaling elements and pathogenic elicitors in a cell-to-cell manner [5, 6]. The levels of callose at PD are controlled by two groups of enzymes, callose synthases and beta-1,3 glucanases, which synthesize and degrade callose, respectively [7]. A number of studies have shown that resistance-inducing chemicals, such as abscisic acid (ABA) [8, 9], salicylic acid (SA) [10], auxin [11], and reactive oxygen species (ROS) [12], contribute to the regulation of the callose balance at PD. The previous studies showed that callose deposition in the neck of PD and phloem plays key roles in restricting virus transportation between cells and long-distance transportation of SMV in soybean, respectively [13, 14]. Additionally, as an intracellular signaling molecule, nitric oxide (NO), which acts upstream of hydrogen peroxide (H_2_O_2_), was reported to play synergistic roles during SMV-induced callose deposition in soybean [15, 16]. Therefore, callose deposition is believed to play a critical role in preventing virus transportation between cells as a physical barrier.

The WRKY family, which is involved in various response pathways to biotic and abiotic stress [17, 18], constitutes one of the ten largest TF families and exists exclusively in higher plants. Members of the WRKY family have been identified in many species, such as *Arabidopsis thaliana* [19], *Nicotiana tabacum* [20], *Zea mays* [21], *Triticum aestivum* [22], *Cymbidium sinense* [23], and *Akebia trifoliata* [24]. WRKY TFs usually contain a highly conserved WRKY domain at the N-termini and a zinc finger-like motif at the C-termini [17]. As the representative domain of the WRKY TFs, the WRKY domain shows sequence variation across plant species, such as WRKYGQK, WRKYGEK, and WRKYGKK [25]. WRKY domains are necessary for WRKY TFs to recognize and bind to the W-box cis-elements (TTGACC/T) of their target genes [26]. Two main types of the zinc finger-like motifs have been identified in WRKY TFs, namely, C2H2 (C-X4-5-C-X22-23-HXH) and C2HC (C-X7-C-X23-HXC) [27]. WRKY TFs can be categorized into three groups (I, II, and III) based on the number of the WRKY domains and the types of zinc finger-like motifs [28]. WRKY TFs in Groups I and II harbor two and one WRKY domain, respectively, but all have a C2H2 type zinc finger-like motif; while those in Group III contain one WRKY domain and a C2HC type zinc finger-like motif [25]. Furthermore, Group II WRKY TFs can be divided into five subgroups according to phylogenetic analysis, including IIa, IIb, IIc, IId, and IIe [29].

Several studies have shown that a large number of *WRKY* genes can be induced by defense-related phytohormones, low temperature, and pathogens, participating in pattern-triggered immunity (PTI) and effector-triggered immunity (ETI) through regulating ROS production [30]. These results indicate that *WRKY* genes play important roles in the immune response to biotic and abiotic stress [31]. For example, CmWRKY15-1 interacted with CmNPR1 to promote the expression of the pathogenesis-related genes, resulting in enhanced resistance to chrysanthemum white rust through the SA pathway in chrysanthemum [32]. In *Malus domestica*, *MdWRKY75e* and *MdWRKY100* overexpression enhanced resistance to *Colletotrichum gloeosporioides* and *Alternaria alternata* [33, 34]. In cotton, the overexpression of *GhWRKY41* enhanced resistance to *Verticillium dahliae* by regulating phenylpropanoid metabolism [35]. In tobacco, the overexpression of the cotton *GhWRKY15* enhanced its resistance to *tobacco mosaic virus* (TMV) and *cucumber mosaic virus* (CMV) by reducing ROS accumulation [36]. In *Arabidopsis*, AtWRKY8 improves plant defense against TMV by modulating regulators that are involved in the ABA and ethylene (ET) signaling pathways such as *ABI4*, which inhibits the expression of *ACS6* and *ERF104* [37].

In soybean, the identification of the WRKY TFs has been reported under both biotic and abiotic stress. For example, Yin et al. (2013) reported 133 GmWRKYs based on Glyma1 assembly (Wm82.a1.v1) and revealed that the large GmWRKY TF family was expanded by segmental duplication events and subsequent divergent selection among subgroups [38]. Bencke-Malato et al. (2014) reported 182 GmWRKYs that included 33 putative pseudogenes based on Glyma1 assembly (Wm82.a1.v1) and analyzed the members in response to *P. pachyrhizi* [39]*.* Song et al. (2016) reported 176 GmWRKYs using the soybean genome (Wm82.a2.v1) and analyzed the expression pattern of *GmWRKYs* under dehydration stress and salt stress [40]. Yu et al. (2016) identified 188 GmWRKYs based on the assembly v2.0 in Phytozome 10.2 and revealed 3 GmWRKYs that are no longer found in existing soybean genomes. In addition, 35 *GmWRKY* genes were identified and showed decreased expression levels under salt stress [41]. Yang et al. (2017) reported 174 GmWRKYs and analyzed the members that promote resistance to soybean cyst nematode [42]. Dong et al. (2019) analyzed soybean *WRKY* genes in response to *Peronospora manshurica* infection through transcriptome analysis and found that 22 *WRKY* genes were differentially expressed in resistant versus susceptible genotypes [43]. In these previous studies, *GmWRKY164* was reported to be differentially expressed under salt or SA treatment [40–42], but the function and molecular mechanism underlying are unclear.

In this study, 185 soybean *WRKY* genes were characterized genome-wide, among them, 60 *GmWRKY* genes were differentially expressed during SMV infection according to the transcriptome data. Notably, *GmWRKY164* exhibited high expression levels in the incompatible combination in Jidou 7 infected with SMV strain N3, and the silencing of *GmWRKY164* led to reduced callose deposition and enhanced virus spread upon SMV infection by directly binding to *GmGSL7c* promoter and regulating its expression. Taken together, these results provide a fundamental understanding of the molecular mechanism underlying SMV infection and new clues for further research into the functions of soybean *WRKY* genes.

## Materials and methods

### Soybean planting and inoculation

The SMV strains (N3 and SC8) used in this study were acquired from Dr. Haijian Zhi (Nanjing Agricultural University, China). The soybean variety Jidou 7 was acquired from Prof. Mengchen Zhang (Institute of Cereal and Oil Crops, Hebei Academy of Agriculture and Forestry Sciences, China). Jidou 7 is resistant to the SMV strain N3 (to form an incompatible combination) and susceptible to the SMV strain SC8 (to form a compatible combination) [15]. The soybean variety Nannong 1138–2 was acquired from Dr. Haijian Zhi (Nanjing Agricultural University, China), which is susceptible to both SMV strain N3 and SC8. Soybean seedlings were planted in pots and placed in greenhouses. The temperature was 25℃, and a 14/10 (day/night) photoperiod was applied with a high-pressure sodium lamp as the light source.

The leaf sap from SMV-infected Nannong 1138–2 plants were mixed with emery and gently applied on the soybean leaves of the Jidou 7 plants or Nannong 1138–2 plants using a brush, according to the previous report [16]. Two-week-old seedlings were harvested at 0, 4, 12, 24, and 48 h after inoculation with SMV strains N3 and SC8, respectively. The leaves without veins were immediately frozen in liquid nitrogen and stored at -80℃ for further experiments.

### Identification of the *WRKY* genes in soybean

The latest version of the soybean genome file was downloaded from the Ensembl Plant (https://plants.ensembl.org/index.html). The pfam seed model WRKY (PF03106) was used for building the Hidden Markov Model (HMM) file using HMMER3 server with e-values lower than 0.01. To verify the reliability and exclude false positives, the presence of WRKY domains were confirmed using the SMART database (http://smart.embl-heidelberg.de) and the CDD database (https://www.ncbi.nlm.nih.gov/cdd). In addition, *GmWRKY* transcription factor sequences were downloaded from PlantTFDB (http://planttfdb.gao-lab.org/) and compared with our data.

### Gene collinearity, duplication events and *cis*-element analysis

The genome data of soybean, rice, and *Arabidopsis* were downloaded from the Ensembl database and the syntenic analysis maps were generated using MCScanX and Circos. All *GmWRKY* genes were classified into five different categories, namely, singleton, dispersed, proximal, tandem, and WGD/segmental by the duplicate gene classifier tool in the Multiple Collinearity Scan toolkit (MCScanX) program [44]. The *cis*-acting elements were analyzed by the PlantCARE software using the 2000 bp sequences upstream of the start codon (ATG) in the *GmWRKY* genes.

### Total RNA isolation, transcriptome data, and RT-qPCR

Total RNA was extracted using RNA extraction kit (UNlQ-10 Column TRIzol Total RNA Isolation Kit, Sangon Biotech, Shanghai, China), and the synthesis of the first-strand cDNA was carried out with PrimeScript RT Reagent Kit with gDNA Eraser (TaKaRa, Dalian, China). In our previous studies, H_2_O_2_-associated transcriptome data were obtained under imidazole (an NADPH oxidase-specific inhibitor) treatment at 0, 4, 12, 24, and 48 h post-SMV N3 inoculation (hpi) [16]. The soybean leaves that were pre-injected with dH_2_O were used as control. RNA samples of the three independent biological replicates were mixed in equal amounts for the construction of libraries. Transcriptome libraries were sequenced on a sequencer (HiSeq 2000, Illumina Inc., San Diego, CA, USA) using 90-base pair-ended modes [16]. Differentially expressed genes were defined as genes with false discovery rate (FDR) less than 0.001 and two-fold change between iminazole and ddH_2_O treatment at each time point [16]. The expression levels of the 60 differentially expressed *GmWRKYs* in response to SMV infection in Jidou 7 according to the transcriptome data was visualized using TBtools [45].

Quantitative reverse transcription PCR (RT-qPCR) was performed to confirm the response of differentially expressed *GmWRKY* genes to SMV infection. *GmEF1b* was used as the internal control. The primers used are listed in Table S1. The RT-qPCR amplification was performed on a Roche LightCycler 96 machine using TransStart® Tip Green qPCR SuperMix (TransGen Biotech, AQ141-01) as the fluorescent dye. The PCR assay was carried out in a total volume of 10 µL, containing 5 µL of 2xTransStart Tip green qPCR supermix, 1 µL of the diluted cDNA, 0.5 µL of each primer (10 µM), and 3 µL of the sterile distilled ddH_2_O. The cycle conditions were described as follows: 94℃ for 30 s, followed by 35 cycles of 94℃ for 5 s, 55℃ for 15 s and 72℃ for 10 s. Amplification curve analysis of the amplification products at the end of each thermal cycling reaction was performed to confirm the specificity of the amplification and to ensure successful amplification and detection, and the relative fold change in the target gene expression was calculated using the 2-^ΔΔ^Ct method [46]. Significant differences were indicated by different lowercase letters, as determined by the LSD test at *p* < 0.05.

### Generation of *GmWRKY164*-silenced plants

*Tobacco rattle virus* (TRV) mediated virus-induced gene silencing (VIGS) was used to generate *GmWRKY164*-silenced plants. The specific fragment of *GmWRKY164* was amplified from the cDNA of Jidou 7 using the primers listed in Table S1 and inserted into pTRV2 plasmid between *Bam*H I and *Xho* I. The empty plasmid of pTRV2 was used as the control (TRV:*00*). *Agrobacterium tumefaciens* carrying pTRV1 and the recombinant pTRV2 (TRV:*GmWRKY164*) were resuspended in the infection buffer (50 mM MES, 2 mM Na_3_PO_4_, 28 mM D-glucose, 0.1 mM acetosyringone, 4.1 mM L-Cys, and 0.02% (w/v) Silwet L-77) until the cell density (OD600) reached 0.5. Then, the mixture of the resuspended pTRV1 and the recombinant pTRV2 (1:1) was poured into the roots of the soybean seedlings for each plant. Before SMV inoculation, the silencing efficiency of GmWRKY164 in the first compound leaves of soybean seedlings was quantified by RT-qPCR three weeks after *A. tumefaciens* treatment. The VIGS experiment and the calculation of silencing efficiency were conducted as the previous reported [16, 47].

### Observation of SMV-induced callose and the detection of SMV *CP* transcript products

The soybean leaves were collected at 48, 72, and 120 h after SMV infection for aniline blue staining and fluorescence observation. The soybean leaves were placed in a fixative solution (50% ethanol, 16.67% glycerol, 16.67% phenol, and 8.33% lactic acid, v/v) and boiled for 2 min, washed for 5 min with ddH_2_O three times, and then treated with the dye solution (0.01% aniline blue dissolved in 0.1 M PBS, pH 7.0) for 15 min. The leaves were washed for 5 min with ddH_2_O 3 times again and observed with a fluorescence microscope (BX53, Olympus, Tokyo, Japan) with Ex/Em = 385 nm/495 nm. ImageJ [48] was used to analyze the microscopic images of the fluorescence of callose and the callose area of 30 inoculation sites was quantified. The upper leaves were observed 15 days after SMV inoculation. According to the previous reported [47], the expression of SMV *CP* was detected using Taq DNA polymerase using semi-quantitative reverse transcription polymerase chain reaction (RT-PCR). RT-qPCR was performed to confirm the expression of SMV *CP* gene with three biological replicates, and *GmEF1b* was used as the internal control. Each biological replicate contains three plants. Values of 0, 4, 12, 24, and 48 indicate hours post inoculation (hpi). The primers used are listed in Table S1.

### Chromatin immunoprecipitation (ChIP) and ChIP-qPCR

ChIP assays were carried out as the previous described [49]. The leaves of Jidou 7 were infiltrated with *Agrobacterium tumefacien* (GV3101) containing 35S:GmWRKY164-GFP or 35S: GFP. 2 g three-week-old leaves were sampled at 40 hpi with N3 and crosslinked with 1% (w/v) formaldehyde for 30 min under vacuum and quenched with 2.0 M glycine for 5 min. The chromatin was sheared to 100–500 bp fragments via ultrasonic disruption (Diagenode, Belgium). Immunoprecipitation was performed with Dynabeads Protein G (Sigma-Aldrich, Germany) and anti-GFP antibody (Novus, USA). For the ChIP-qPCR assay, the amount of immunoprecipitated *GmGSL7c* chromatin in the P1 and P2 regions on its promoter was detected by qPCR. The primers used are listed in Table S1.

### Electrophoretic mobility shift assay

Electrophoretic mobility shift assay (EMSA) was performed using the Light Shift Chemiluminescent EMSA Kit (Thermo Fisher Scientific, USA). The double-stranded DNA probe containing W-box was labeled with biotin at 5’ end (Table S1). Nonlabelled probes (200-fold) were used as competitors. The biotinylated probes (Table S1) were synthesized by Sangon Biotech (Shanghai, China). The biotinylated and unlabeled probers were incubated with 6 µg of His-tagged GmWRKY164 protein in binding reactions (provided with the EMSA kit) for 20 min. The reactions were stopped by the addition of loading buffer and the proteins were resolved on a 6% (w/v) native polyacrylamide gel. Then, the complex was electrophoretically transferred to a Hybond-N^+^ nylon membrane (Solarbio, China), which was subsequently crosslinked under ultraviolet light. The signals were detected using a charge-coupled device camera.

## Results

### Characteristics of the *GmWRKY* genes in soybean

**Fig. 1:**
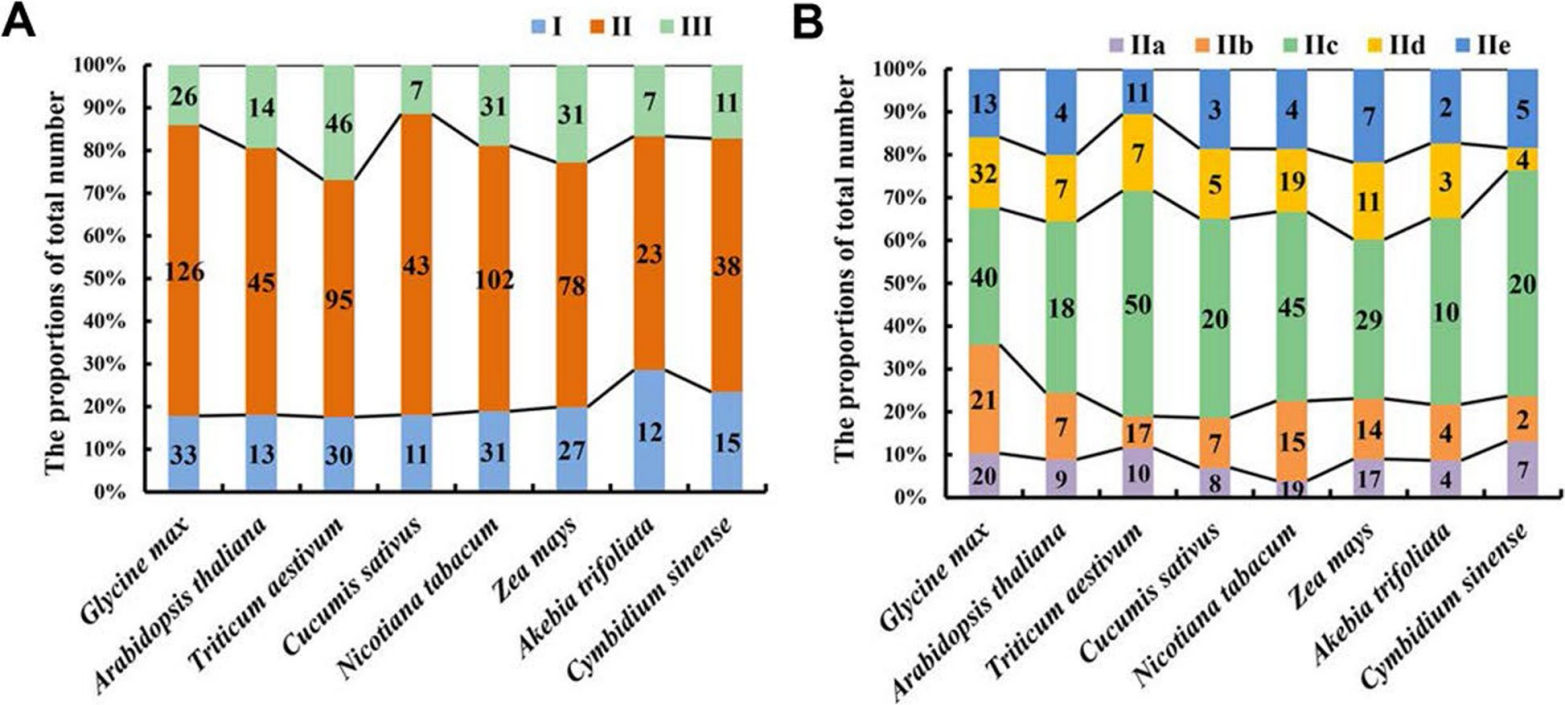
Statistics on the number of *WRKY* genes in eight plant species. **A** The number of *WRKY* genes in Group I, Group II and Group III in eight plant species. **B** The number of *WRKY* genes in Groups IIa, IIb, IIc, IId and IIe in eight plant species

A total of 185 GmWRKY (Table S2) members were annotated in the latest version of the soybean genome file (Glycine_max_v2.1), which is consistent with the number recorded in the PlantTFDB. The 185 *GmWRKYs* were named according to the report by Yu et al. (2016) [41]. Then we investigated the number and proportion of WRKY members in eight representative plant species, including *Glycine max*, *Arabidopsis thaliana*, *Triticum aestivum*, *Cucumis sativus*, *Nicotiana tabacum*, *Zea mays*, *Akebia trifoliata*, and *Cymbidium sinense* (Table S3 and Fig. 1). The number of *WRKY* genes in *Glycine max* far exceeded those in other species, except for *Triticum aestivum* and *Nicotiana tabacum*, which are both allopolyploid plant species. The proportions of GmWRKY proteins in Group I were generally the same in different species except for *Akebia trifoliata* and *Cymbidium sinense*. The number of WRKY proteins in Group II was generally greater than that in Group I and Group III in the eight plant species, ranging from 54.8% to 70.5%. Additionally, Group IIc had the most WRKY members in the eight representative plant species. These results suggested that the majority of members of Group II might experience a significant expansion in soybean and other species, which could be caused by whole-genome duplications, tandem duplications, or segmental duplications of genomes.

### Duplication events and syntenic analysis of the GmWRKY members

**Fig. 2:**
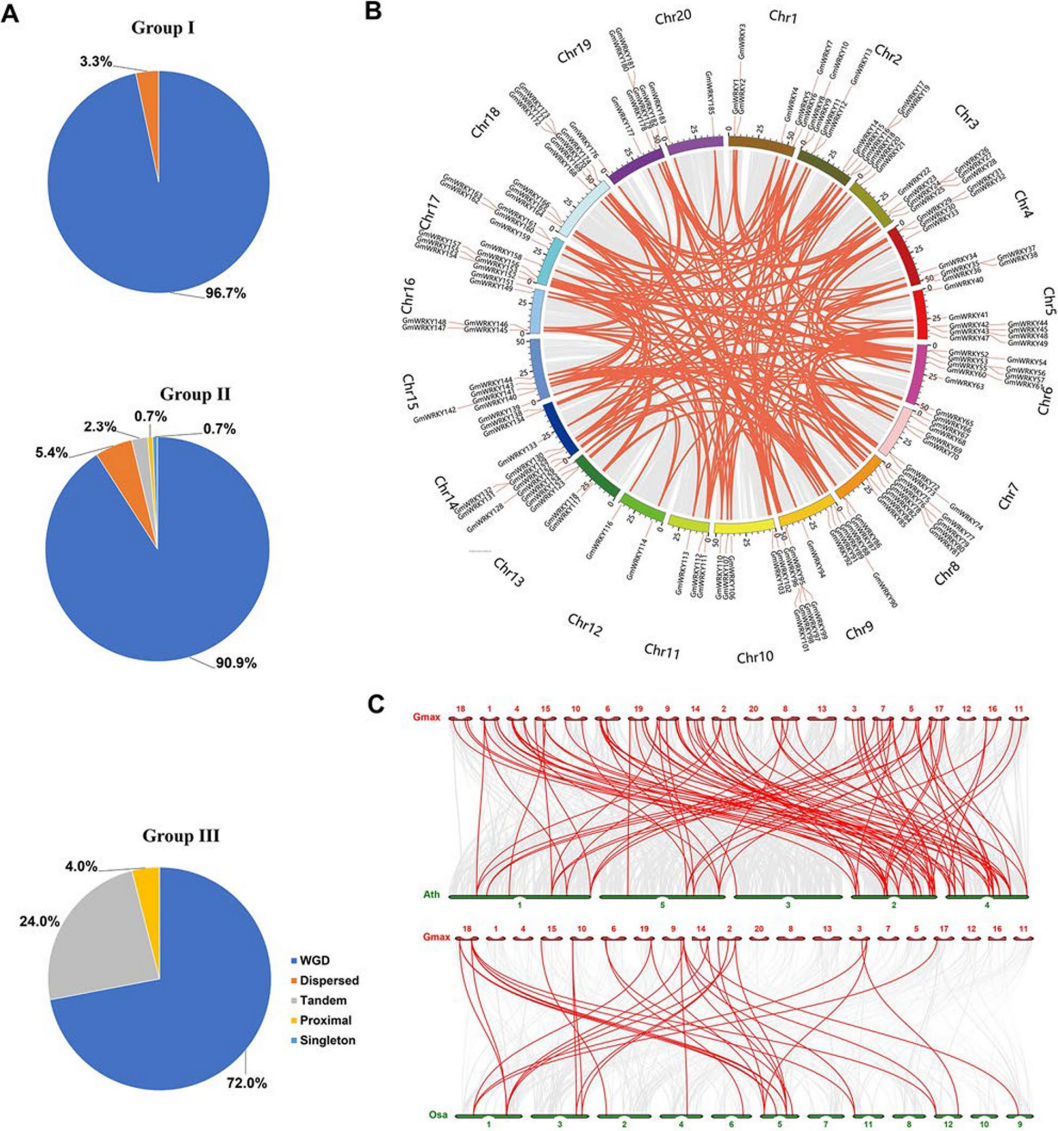
Duplication events and syntenic analysis of *GmWRKYs*. **A** The proportion of *GmWRKY* genes that exhibit different modes of duplication in the soybean genome. **B** Chromosome distribution and collinearity of duplicated *GmWRKY* pairs. Chromosomes are represented by differently colored boxes. **C** Collinearity analysis of *WRKY* family genes between soybean and *Arabidopsis*, soybean and rice, respectively. The gray lines indicate the collinear pairs in the soybean, rice, and *Arabidopsis* genomes, while the red lines highlight the collinear WRKY pairs. Gmax, Osa, and Ath indicate soybean, rice, and *Arabidopsis*, respectively

Two diploid soybean species experienced two sequential WGD events, including a polyploidy event at ~ 59 MYA and a Glycine-specific WGD at ~ 8–13 MYA [50, 51]. Duplicated genes in soybean have been classified into five categories, including singletons, dispersed, proximal, tandem, and WGD (including segmental duplications), which strongly correlate with gene function [50]. Therefore, we analyzed the duplication types of *GmWRKY* genes via MCScanX (Fig. 2). The results showed that WGD events accounted for 96.7% (29/30), 90.9% (118/130), and 72.0% (18/25) in Groups I, II, and III, respectively (Fig. 2A). In addition, dispersed duplication events accounted for 3.3% (1/30) and 5.4% (7/130) in Group I and II, respectively. Tandem and proximal duplication events occurred mainly in Group III, accounting for 24.0% (6/25) and 4.0% (1/25), respectively. WGD events were subsequently visualized within the soybean genome, and 220 segmental duplication pairs were found between 165 *GmWRKY* genes (Fig. 2B and Table S4). The Ka/Ks (nonsynonymous/synonymous substitutions) of 220 WGD gene pairs were calculated to be less than 1 (Table S4), indicating that the expansion of *GmWRKYs* was caused mainly by the WGD events under negative selection pressure during their evolution. The synthetic analysis of *WRKYs* in *Arabidopsis* and rice were performed with *GmWRKYs* (Fig. 2C). The result showed that 96 orthologous pairs were identified according to the whole-genome-wide comparative analysis between soybean and *Arabidopsis*, and 33 orthologous pairs were identified between soybean and rice (Table S5). Among them, 76 and 19 *GmWRKYs* (41.1% and 10.3%) displayed syntenic relationships with 35 and 17 *AtWRKYs* and *OsWRKYs* (48.6% and 17.0%) in *Arabidopsis* and rice, respectively*.*

### Promoter analysis of the *GmWRKY* genes

**Fig. 3:**
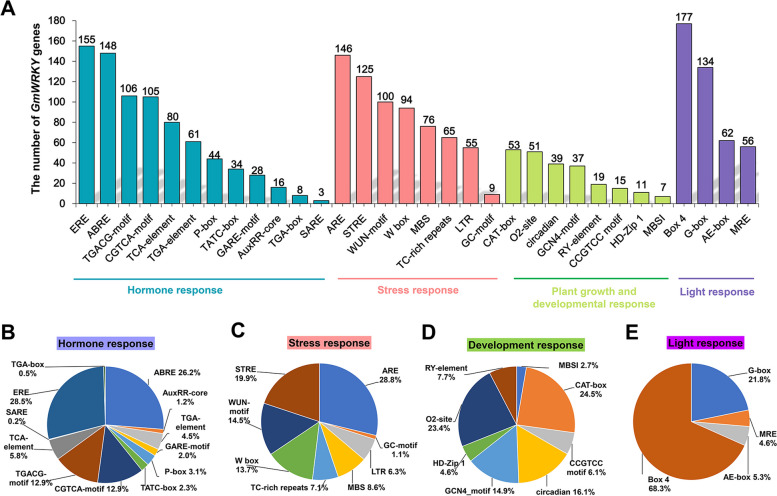
Types and numbers of cis-acting elements in promoters of *GmWRKY* genes. **A** The number of each type of cis-acting element that is present in *GmWRKYs*. The proportion of each cis-acting element based on its biological function, including hormone-responsive elements (**B**), stress-responsive elements (**C**), cis-acting elements involved in plant development and growth (**D**), and light-responsive cis-acting elements (**E**)

To explore the regulatory mechanisms of *GmWRKYs*, 32 types of cis-elements were identified in the promoters of *GmWRKYs* [52, 53], including those in hormone responses, stress responses, plant growth and developmental responses, and light responses (Fig. 3). Among them, 12 hormone-responsive elements were found, including Abscisic Acid (ABRE), Auxin (AuxRR-core, TGA-element and TGA-box), Gibberellin (GARE-motif, P-box and TATC-box), MeJA (CGTCA-motif and TGACG-motif), Salicylic Acid (TCA-element and SARE), and Ethylene (ERE). Among the hormone-responsive elements, the ERE and ABRE were widely distributed among the promoters of *GmWRKYs* (Fig. 3A) and occupied the largest proportions (28.53% and 26.25% respectively) (Fig. 3B). Eight stress-responsive related elements (Fig. 3C) were identified, including ARE (anaerobic induction), GC-motif, LTR (low-temperature), MBS (drought), TC-rich repeats (stress-responsive), W-box, WUN-motif (wound), and STRE (heat shock response). Remarkably, the W-box existed in 94 *GmWRKYs*, and can be specifically recognized and bound by WRKY TFs, indicating that these *GmWRKYs* may be regulated by themselves or other WRKY TFs at the transcriptional level. Eight elements were involved in plant growth and developmental responses (Fig. 3D), including MBSI (MYB binding site), CAT-box (meristem-specific element), CCGTCC-motif (meristem-specific activation), GCN4-motif (endosperm-specific element), circadian (circadian control responsiveness), HD-Zip 1 (palisade mesophyll cells differentiation), O2-site (zein metabolism regulation), and RY-element (seed-specific regulation). Four elements involved in light response were detected (Fig. 3E), including Box 4, G-box, AE-box and MRE. Among them, the Box4 elements exhibit a wide distribution in *GmWRKY* genes, which were identified in 177 (95.7%) members of *GmWRKYs* and occupied the largest proportions among light response-related motifs (68.33%), providing a reference for further studies.

### Expression patterns of the *GmWRKYs* during SMV infection

**Fig. 4:**
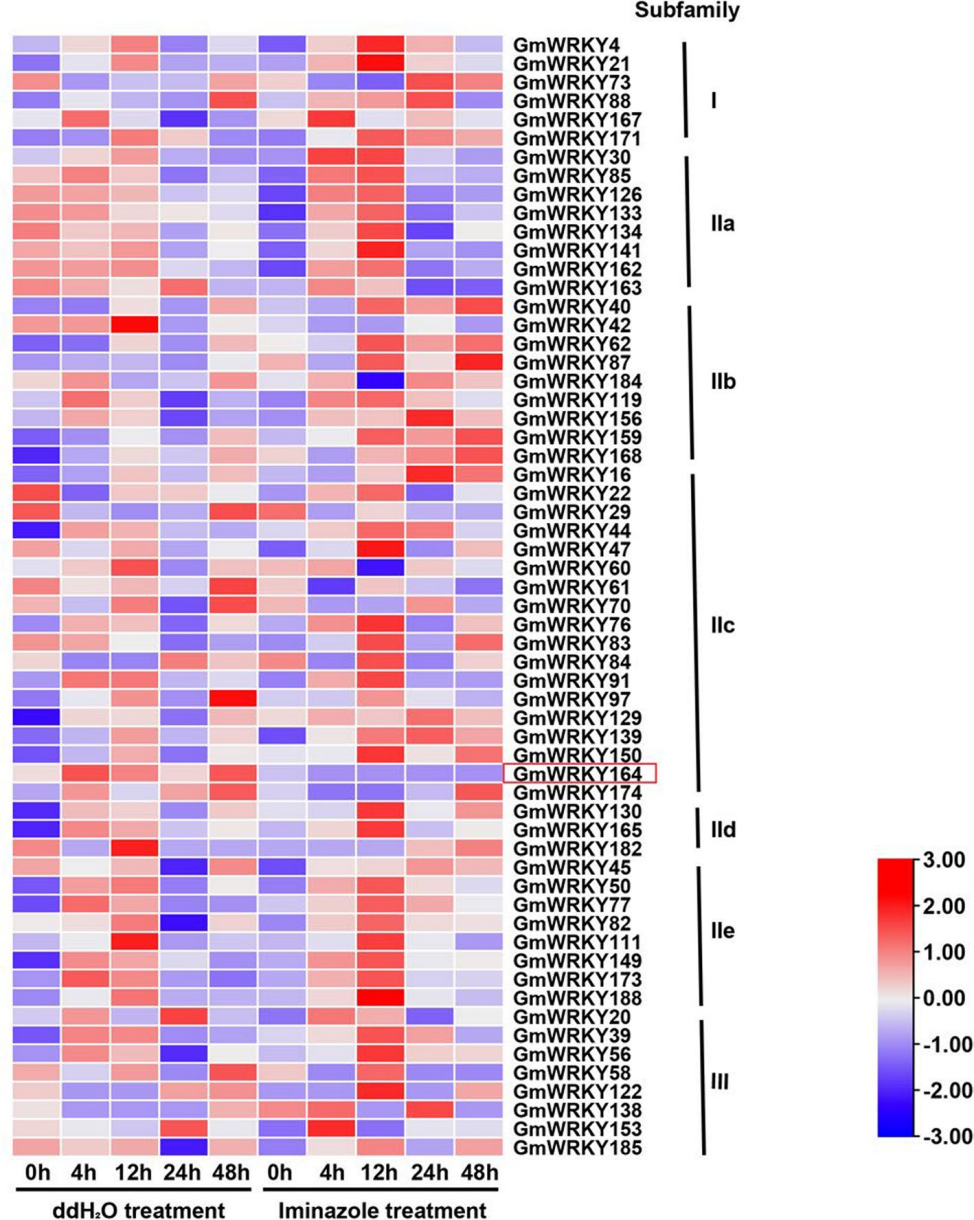
The expression levels of 60 differentially expressed *GmWRKYs* in response to SMV infection in Jidou 7 according to the transcriptome data. *GmWRKY164* (*Glyma.17G224800*) is outlined in red. The hour points indicate 0, 4, 12, 24, and 48 h after SMV infection under water (the control) and imidazole treatments. Differentially expressed genes were defined as genes with false discovery rate (FDR) less than 0.001 and two-fold change between iminazole and ddH_2_O treatment at each time point. The result was visualized using TBtools [45]

**Fig. 5:**
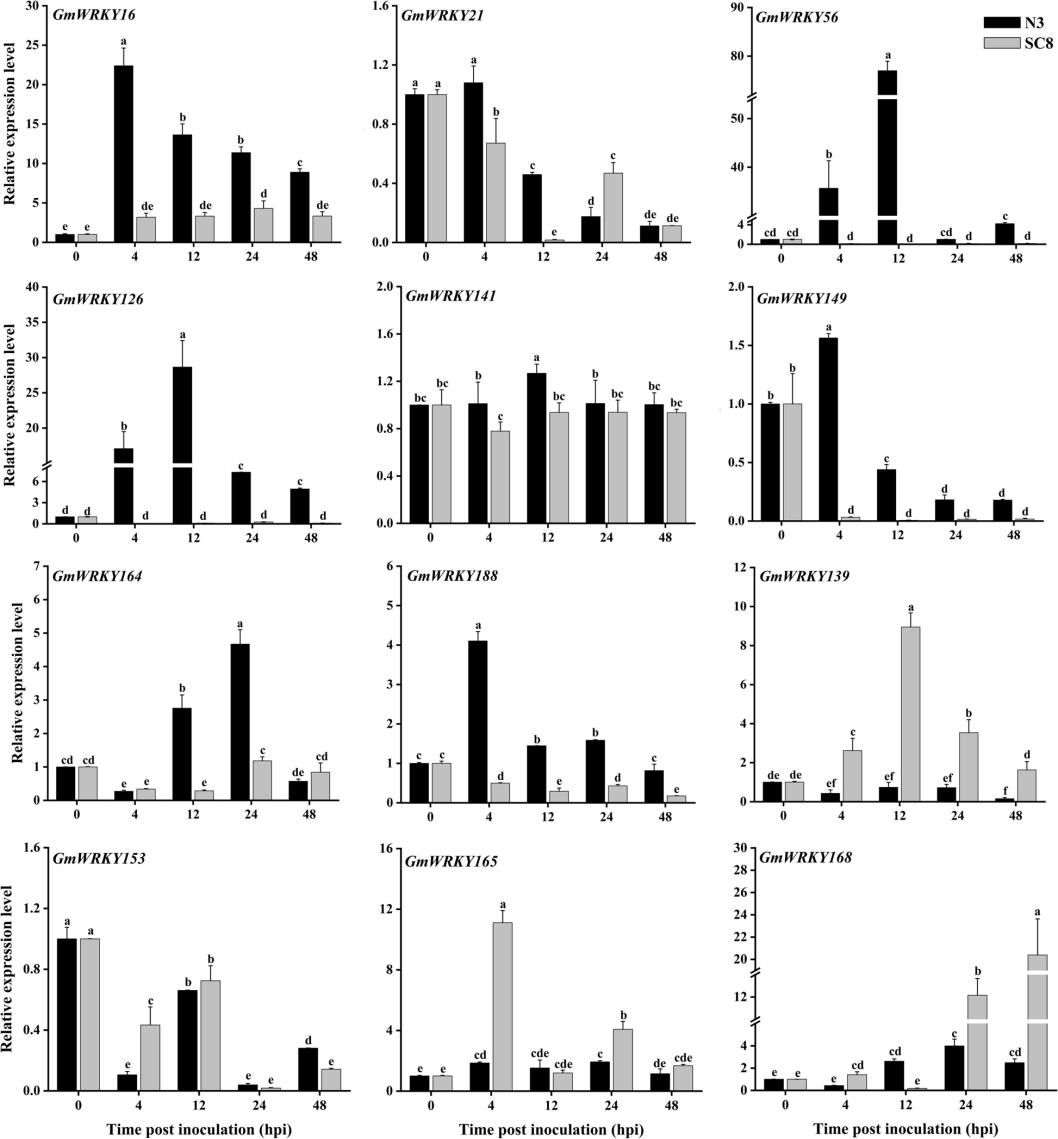
The expression levels of differentially expressed *GmWRKYs* in response to SMV infection. The soybean cultivar variety Jidou 7 is resistant and susceptible to SMV strains N3 and SC8 respectively. Values of 0, 4, 12, 24, and 48 indicate hours post inoculation (hpi). Each experiment was performed with three biological replicates. Each biological replicate contains three plants. The data is represented as mean ± SD (*n* = 3). Significant differences were indicated by different lowercase letters, as determined by the LSD test at *p* < 0.05

In our previous studies, we found that NO and H_2_O_2_ played synergistic roles in callose deposition during SMV infection, which is crucial for restricting virus transportation [15, 16]. To investigate the roles of *GmWRKYs* in response to SMV infection, we identified 60 *GmWRKY* genes (Fig. 4) that were differentially expressed upon SMV infection according to H_2_O_2_-associated transcriptome data [16]. Among them, 7 and 8 *GmWRKYs* belonged to Group I and Group III, respectively. In addition, 45 members belonged to Group II, including 8 in Group IIa, 9 in Group IIb, 18 in Group IIc, 3 in Group IId, and 8 in Group IIe (Fig. 4). Furthermore, 12 *GmWRKYs* were selected in Groups and were subjected to RT-qPCR analysis for the validation of their expression patterns upon SMV infection (Fig. 5). The results showed that some genes, such as, *GmWRKY16*, *GmWRKY56*, *GmWRKY126*, *GmWRKY141*, *GmWRKY149*, *GmWRKY164*, and *GmWRKY188*, were highly expressed in the incompatible combination (Fig. 5). However, *GmWRKY139*, *GmWRKY165*, and *GmWRKY168*, were highly expressed in the compatibility combination. Among them, *GmWRKY164* in Group IIc, which had high expression levels in the incompatible combination and was downregulated after imidazole treatment (Fig. 4 and Fig. 5), was selected for further functional analysis.

### GmWRKY164 plays a positive role in resistance to *soybean mosaic virus*

**Fig. 6:**
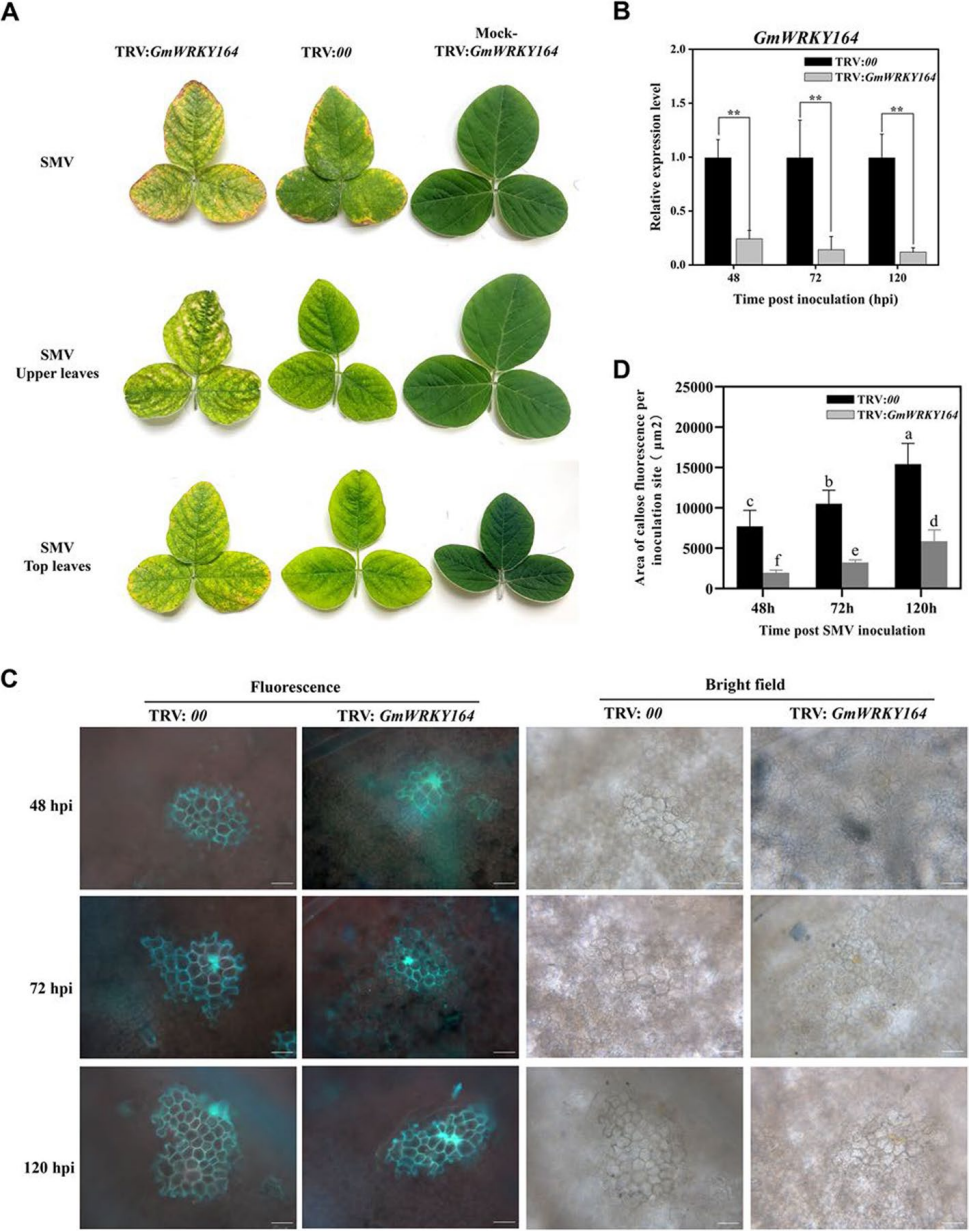
Silencing of *GmWRKY164* reduces callose deposition in Jidou 7 during SMV infection. **A** Phenotypes of inoculated leaves, non-inoculated upper and top leaves in *GmWRKY164*-silenced plants inoculated with SMV N3 at 15 dpi. The Mock-TRV:*GmWRKY164* indicates the TRV:*GmWRKY164* without SMV inoculation. **B** Relative expression of *GmWRKY164* in the *GmWRKY164-*silenced plants in Jidou 7 after SMV N3 inoculation. The relative expression level of *GmWRKY164* in TRV:*00* at each time point was defined as 1. Each experiment was performed with three biological replicates. Each biological replicate contains three plants. The significance of all comparisons was determined using Student’s *t* test, *p < 0.05, **p < 0.01. **C** Callose was observed on the leaves of *GmWRKY164*-silenced plants inoculated with SMV N3 after 48, 72 and 120 h. The callose was stained with aniline blue and observed under the fluorescence microscope (Olympus BX53). Bar = 50 μm. **D** The area of callose fluorescence per inoculation was analyzed for the *GmWRKY164*-silenced plants in Jidou 7 after SMV N3 inoculation. The presence of at least 30 discontinuous infestation sites was calculated. Values are mean ± SD of 30 independent measurements. Representative images of three biological replicates are shown in C. Lowercase letters indicate significant differences between different samples by one-way ANOVA test followed by Duncan's range test (*p* < 0.05)

To investigate the role of GmWRKY164 in response to SMV, the *GmWRKY164*-silenced plants were generated via TRV-mediated VIGS in Jidou 7 with the silenced efficiency of more than 50% determined by RT-qPCR. Compared to the control (TRV:*00*), the disease symptoms in the inoculated, non-inoculated upper and top leaves at 15 dpi (Fig. 6A and Fig. 6B) of the *GmWRKY164*-silenced plants displayed severe mosaic symptoms with more chlorosis and necrotic spots. The area of cells with callose fluorescence induced by SMV infection was significantly decreased at 48, 72, and 120 hpi in *GmWRKY164*-silenced plants (Fig. 6C and 6D). In addition, the SMV-susceptible cultivar Nannong 1138–2 infected with N3 and SC8 showed typical mosaic symptoms on the upper leaves, especially for SC8, indicating that SC8 has more enhanced virulence than N3 in Nannong 1138–2 (Figure S1A). RT-qPCR analysis indicated that *GmWRKY164* had lower expression levels in both Nannong 1138–2 and Jidou 7 plants infected by SC8 (Figure S1B), exhibiting more pronounced necrosis symptoms than those infected by N3. In addition, *GmWRKY164* displayed higher expression levels at 12 and 24 hpi in Jidou 7 infected by N3 compared to SMV strain SC8 (Figure S1C), confirming that *GmWRKY164* plays a positive role in resistance to SMV. Furthermore, the expression level of the coat protein (*CP*) gene in SMV was significantly increased in Jidou 7 infected by N3 (Figure S2), indicating that the viral load was increased in the *GmWRKY164*-silenced plants upon SMV infection. These results suggested that *GmWRKY164* may play a positive role in response to SMV through increasing callose deposition to inhibit virus spreading.

### GmWRKY164 directly targets *GmGSL7c* to suppress virus spread through increasing callose deposition

**Fig. 7:**
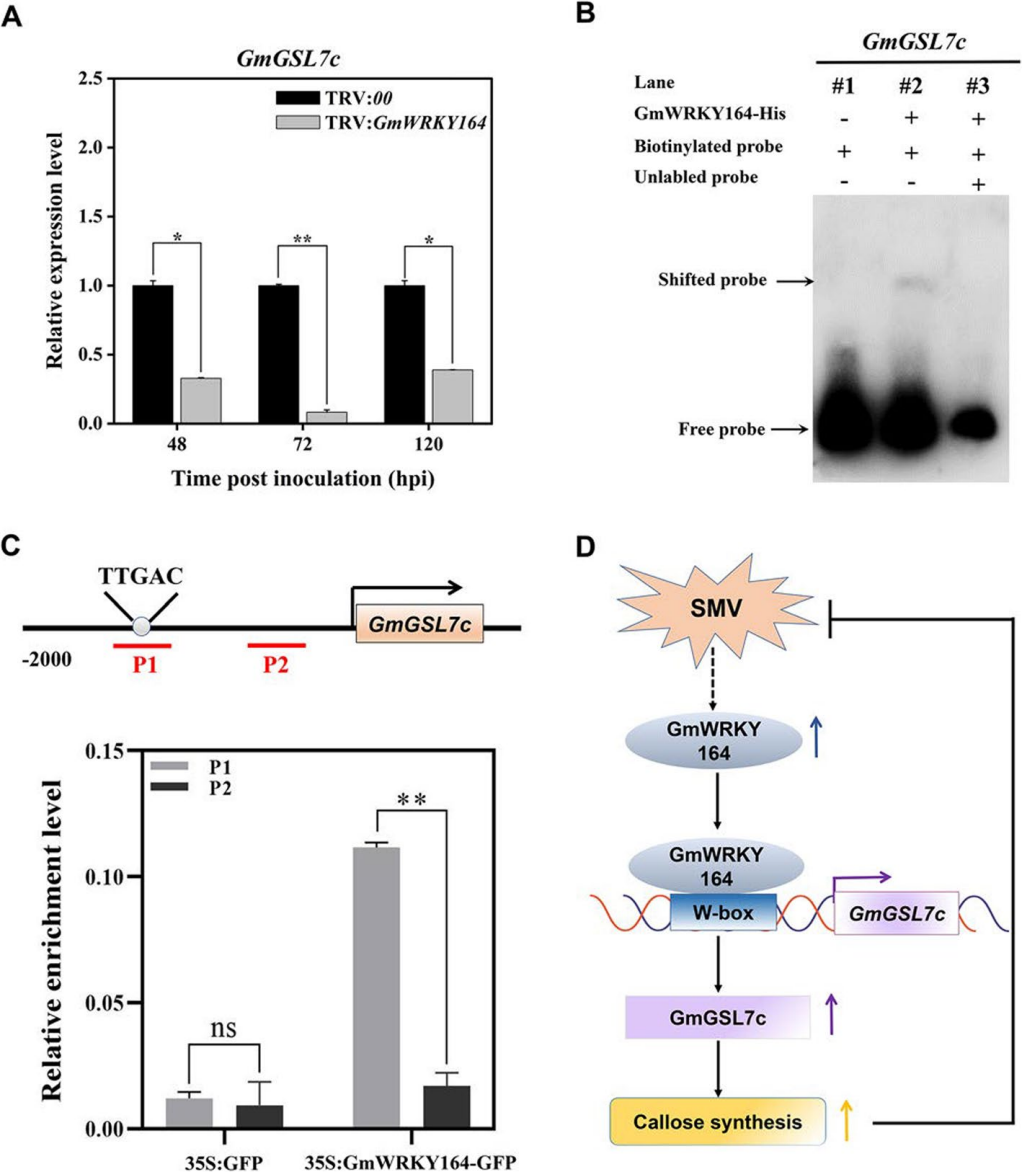
GmWRKY164 binds to the promoter of *GmGSL7c* both *in vitro* and *in vivo*. **A** Relative expression of *GmGSL7c* in the *GmWRKY164*-silenced plants in Jidou 7 after SMV N3 inoculation. The relative expression level of *GmGSL7c* in TRV:*00* at each time point was defined as 1. Each experiment was performed with three biological replicates. Each biological replicate contains three plants. Significance of all comparisons was determined using Student’s *t* test, *p < 0.05, **p < 0.01. **B** EMSA shows that GmWRKY164 binds directly to the W-box (GTCAA) of the *GmGSL7c* promoter. The W-box element was labeled with biotin and used as a probe. Non-labeled probes (200-fold) were used as competitors. Shifted and free probes indicate the complexes of protein-probes and unbound probes, respectively. The uncropped blots are presented in Figure S5. **C** ChIP-qPCR analysis of GmWRKY164 binding to the* GmGSL7c *promoter. Schematic diagrams of *GmGSL7c* promoter. The gray circle indicates the position of the W-box. P1 and P2 indicate the fragments in the *GmGSL7c* promoter used for ChIP-qPCR analysis. Chromatin isolated from 35S:GmWRKY164-GFP and 35S:GFP leaves were immunoprecipitated with anti-GFP antibody. Isolated gDNA was amplified by qPCR, and the results for each ChIP sample was normalized to those for the input samples. Asterisks indicate significant differences by Student's *t*-test (**p < 0.01). **D** Diagram depicting the mechanism of the GmWRKY164-*GmGSL7c* module in response to SMV. Upon SMV infection, the expression level of *GmWRKY164* increases, and the protein it encodes could bind to the W-box and promotes the expression of *GmGSL7c*, thereby resulting in enhanced callose synthesis to suppress SMV spread

To further understand how *GmWRKY164* works during SMV infection, 693 differentially expressed genes (DEGs) that show similar expression trends with *GmWRKY164* were identified according to the transcriptome data (Table S6) [16]. Among them, a glucan synthase-like gene (named *GmGSL7c*, *Glyma.08G308200*) was found to be involved in callose synthesis [54]. In addition, the transcript levels of *GmGSL7c* were dramatically lower in the *GmWRKY164*-silenced plants in Jidou 7 (Fig. 7A). Furthermore, a W-box (GTCAA, -1901 bp to -1905 bp) was found through the prediction of the *cis*-elements in the *GmGSL7c* promoter region (Table S7). To test this hypothesis, electrophoretic mobility shift assay (EMSA) in vitro was performed, which revealed that GmWRKY164 could directly bind to the W-box motif of the *GmGSL7c* promoter (Fig. 7B). In this assay, the biotin-labeled probe containing a combination of the W-box from the *GmGSL7c* promoter was constructed and incubated with the recombinant protein His-tagged GmWRKY164. The mobility shift we observed, indicated that GmWRKY164 bound to W-box motif of the *GmGSL7c* promoter in vitro (Fig. 7B). Next, ChIP-qPCR was used to determine the enrichment of GmWRKY164-GFP in the P1 region (-1947 bp to -1879 bp, containing a W-box motif) and the P2 region (-781 bp to -735 bp, without a W-box motif) of the *GmGSL7c* promoter (Fig. 7C). These results suggested that the enrichment of GmWRKY164-GFP was markedly greater in the P1 region than that in the P2 region in the 35S:GmWRKY164-GFP plants, but not in the 35S:GFP plants (Fig. 7C). In summary, these results demonstrated that GmWRKY164 directly binds to the promoter of *GmGSL7c *in vitro and in vivo to promote its transcription, which increases callose deposition and suppresses virus spreading (Fig. 7D).

## Discussion

Plant WRKY transcription factors are one of the key components of plant immune responses and involved in the regulatory network to resistant pathogens. For example, *SlWRKY75* enhances tomato defenses against *Pst* DC3000 by activating the expression of *SlGH3.3* [55]. The exogenous expression of barley *HvWRKY6* enhances resistance to leaf rust, Fusarium crown rot, and sharp eyespot in wheat [56]. In *Arabidopsis*, AtWRKY50 specifically binds to LS10 region of the *PR1* promoter and interacts with TGAs to synergistically activate *PR1* expression [57]. In addition, AtWRKY50 also plays roles in both SA- and low-18:1-dependent repression of JA signaling [58]. In rice, OsWRKY77 is reported to be a positive regulator of *PR1*, *PR2* and *PR5* expressions and basal resistance to the bacterial pathogen *Pst*DC3000 [59]. In soybean, so many evidences illustrated that GmWRKYs participate in a variety of abiotic stresses, for instance, GmWRKY17 and GmWRKY54 improve soybean drought tolerance [60, 61], GmWRKY21 and GmWRKY81 improve soybean Al tolerance [62, 63], GmWRKY142 and GmWRKY172 enhance cadmium tolerance [64, 65], GmWRKY12 and GmWRKY16 enhance drought and salt tolerance [66, 67]. However, the involvement of GmWRKYs in biotic stresses has been less reported. Except for, GmWRKY31 and GmWRKY40 are reported to play positive roles in response to *Phytophthora sojae* [68, 69]. In our study, we identified 60 *GmWRKY* genes that potentially play roles in response to SMV infection. Furthermore, we revealed that GmWRKY164, which had the highest homology with AtWRKY50 (Figure S6), plays a positive role in response to SMV through increasing callose deposition to inhibit virus spreading by activating *GmGSL7c* expression.

The previous studies have shown that phytohormones, low temperature, pathogens and other factors can induce the expression of *WRKY* genes to participate in various physiological processes in plants [70, 71]. For example, OsARF12 can bind to the AuxRE in the *OsWRKY13* promoter to activate its expression, contributing to the antiviral immune response to *rice dwarf virus* in rice [72]. In cotton, GhTINY2 could promote the expression of *WRKY51* through binding to the ERE in its promoter, thereby improving resistance to *Verticillium dahlia* [73]. In tomato, SIWRKY33 can bind to the TCA element at its own promoter to activate its transcription, and enhance the response to cold stress [74]. In our study, numerous cis-acting elements were identified in the promoters of *GmWRKY* genes, especially elements related to phytohormones, including ABA (ABRE), auxin (AuxRR-core, TGA element), gibberellin (GARE motif, P-box, and TATC-box), ethylene (ERE), MeJA (CGTCA motif and TGACG motif) and salicylic acid (TCA element and SARE). Additionally, many other cis-elements, such as W-box, LTR, and MBS, also occur in the promoters of *GmWRKYs*. These results revealed a series of potential transcription factors that regulate the expression of *GmWRKYs*, which provides the theoretical basis and data resources for the molecular regulatory networks associated with soybean response to SMV, but their regulatory mechanism needs to be further explored.

Numerous studies have shown that the deposition of the callose on the plasmodesmata is the main strategy to against virus invasion [13, 75]. Additionally, H_2_O_2_ also plays important roles in plant immune response [29, 71, 76]. In our previous study, it was shown that the decreased callose deposition on the plasmodesmata, due to the inhibition of H_2_O_2_ production, elevated the diffusion of SMV [16]. The previous studies have also shown that WRKY TFs can directly or indirectly regulate the expression of callose synthase genes to affect callose accumulation. For example, OsWRKY46 and OsWRKY72 can directly bind to the promoter region of *CalS* gene to enhance its transcriptional level and promote callose synthesis in rice [77]. In tobacco, NbWRKY40 can bind to the promoter of SA biosynthesis gene (*ICS1*), thereby indirectly affects the expression of callose synthase gene, leading to callose deposition in PD neck to inhibit virus movement, and positively regulating tobacco resistance to *tomato mosaic virus* infection [78]. In sweet sorghum, SbWRKY22 and SbWRKY65 could enhance plant Al tolerance by promoting callose degradation in the root [79]. In soybean, the GmWRKY family members have not been confirmed to participate in the regulation of callose synthesis. In our study, we found that GmWRKY164 plays a positive role in response to SMV through increasing callose deposition to inhibit virus spreading. Under biotic stress, the accumulation of callose is often triggered in plants. The cell wall permeability is controlled by the deposition of callose in cell wall, plasmodesmata, and sieve pore, forming physical barriers to slow down pathogen invasion [80, 81]. Callose synthase (CalS), which catalyzes the synthesis of callose from UDP-glucose [82], is also known as GSL for GLUCAN SYNTHASE-LIKE. The reported study has shown that the decrease of callose in papillae in the loss-of-function mutants of *GSL5* enhanced the invasion of the powdery mildew in *Arabidopsis* [83]. In cotton, GhCalS5 is involved in response to cotton aphid damage through callose formation [84]. In our previous study, the silencing of *GmGSL7c* promoted the transportation of SMV through decreasing callose deposition, indicating that it plays a positive role in the resistance to SMV [54]. In this study, we further found that GmWRKY164 plays a positive role in resistance to SMV infection by regulating the expression of *GmGSL7c*, resulting in the deposition of callose to suppress viral movement. Our findings provide guidance for future studies in understanding virus-resistance mechanisms in soybean.

## Conclusion

In this study, 185 *GmWRKY* genes were characterized in soybean using the Glycine_max_v2.1, and 60 *GmWRKY* genes were identified to be the potential regulators involved in response to SMV infection. Among them, *GmWRKY164* in Group IIc had high expression levels in the incompatible combination and was downregulated after imidazole treatment. The silencing of *GmWRKY164* reduced callose deposition and enhanced virus spread during SMV infection. In addition, EMSA and ChIP-qPCR revealed that GmWRKY164 can directly bind to the promoter of *GmGSL7c*, which is involved in callose synthesis. Therefore, our findings highlighted the important role of GmWRKY164 in resistance to SMV, demonstrating its involvement in callose deposition and virus spreading restriction.

### Supplementary Information

Additional file 1: Figure S1. The expression levels of GmWRKY164 in soybean cultivar varieties Nannong 1138–2 and Jidou 7 after SMV inoculation. Figure S2. Silencing of GmWRKY164 enhances virus spreading during SMV infection. Figure S3. Amplification of CP gene by RT-PCR separation by agarose gel electrophoresis. Figure S4. Amplification of GmEF1b gene by RT-PCR. Figure S5. The full-length blots of EMSA in Fig. 7B. Figure S6. Phylogenetic analysis of WRKY proteins on orthologous members from soybean, rice, and Arabidopsis.

Additional file 2: Table S1. Primers used in this study. Table S2. The WRKY genes in soybean. Table S3. The number of WRKY genes in each subgroup in the eight plant species. Table S4. The 220 WGD gene pairs of GmWRKY genes in soybean. Table S5. Synteny analysis of WRKY genes between soybean, and Arabidopsis and rice, respectively. Table S6. The differentially expressed genes with the similar expression trends with GmWRKY164. Table S7. Promoter analysis of GmGSL7c.

## Data Availability

The latest version of the soybean genome file was downloaded from the Ensembl database (https://ftp.ensemblgenomes.ebi.ac.uk/pub/plants/release-59/fasta/glycine_max/). The seeds used in this experiment were Nannong 1138–2 and Jidou 7. The SMV strains were N3 and SC8. The datasets supporting the conclusions of this study are included in the article and in additional files.
