# Supplementary material for: A comprehensive analysis of the WRKY family in soybean and functional analysis of GmWRKY164-GmGSL7c in resistance to soybean mosaic virus
Source: BMC Genomics. 2024 Jun 19;25:620. doi: 10.1186/s12864-024-10523-8 (PMC11188170; doi:10.1186/s12864-024-10523-8)
Supplement: Supplementary file 1 — Additional file 1: Figure S1. The expression levels of GmWRKY164 in soybean cultivar varieties Nannong 1138–2 and Jidou 7 after SMV inoculation. Figure S2. Silencing of GmWRKY164 enhances virus spreading during SMV infection. Figure S3. Amplification of CP gene by RT-PCR separation by agarose gel electrophoresis. Figure S4. Amplification of GmEF1b gene by RT-PCR. Figure S5. The full-length blots of EMSA in Fig. 7B. Figure S6. Phylogenetic analysis of WRKY proteins on orthologous members from soybean, rice, and Arabidopsis. [file 12864_2024_10523_MOESM1_ESM.docx]

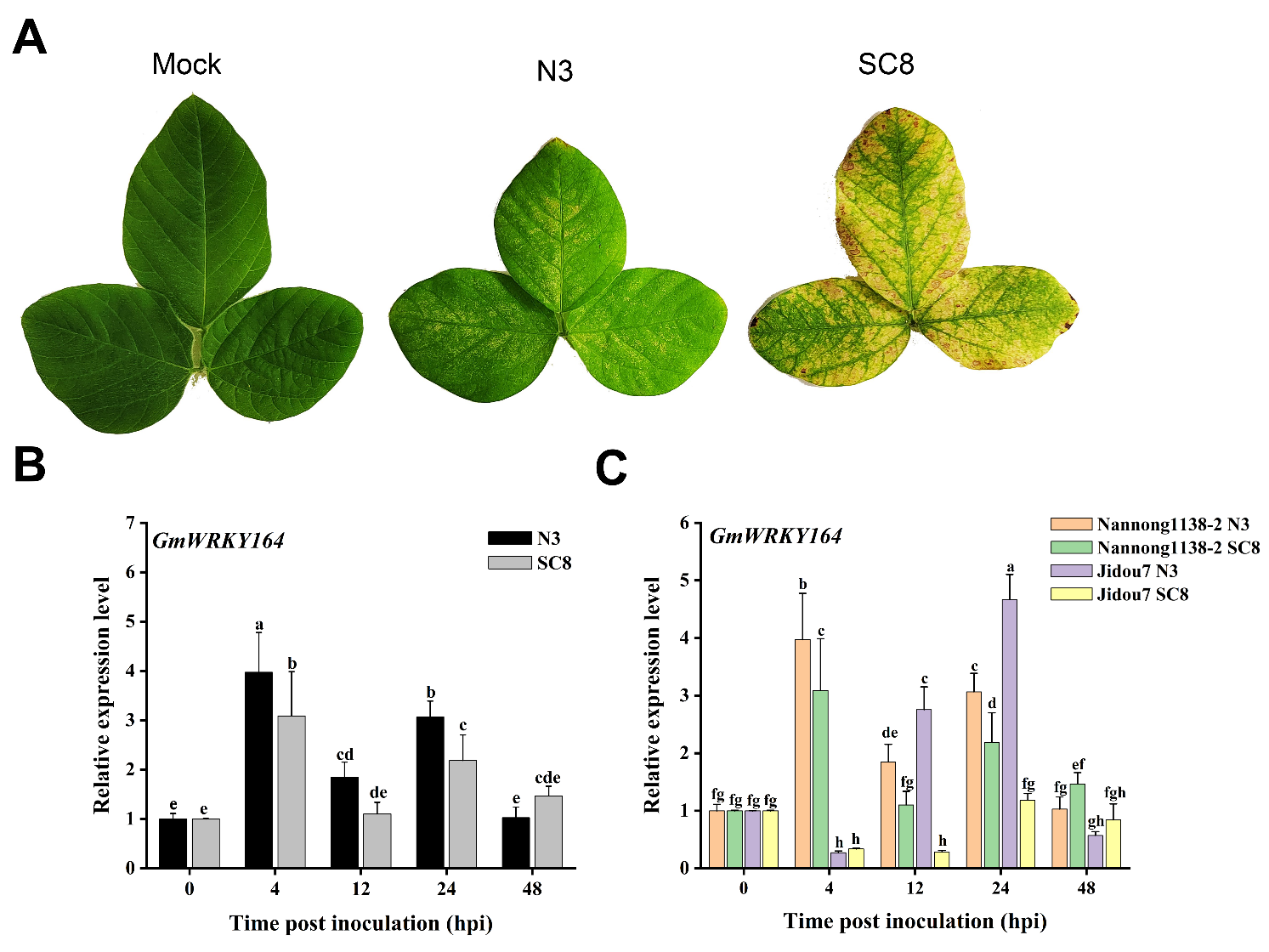


**Figure S1. The expression levels of *GmWRKY164* in soybean cultivar varieties Nannong 1138-2 and Jidou 7 after SMV inoculation.** (A) Soybean cultivar variety Nannong 1138-2 showed typical mosaic symptom 15 days after SMV inoculation. Mock indicates Nannong 1138-2 plants without SMV infection as the negative control. N3 and SC8 indicate Nannong 1138-2 plants infected with SMV strains N3 and SC8. The soybean cultivar variety Nannong1138-2 is susceptible to both SMV strain N3 and SC8. (B) The relative expression of *GmWRKY164* after SMV inoculation in Nannong 1138-2. (C) The relative expression of *GmWRKY164* after SMV inoculation in Nannong 1138-2 and Jidou 7. Values of 0, 4, 12, 24, and 48 indicate hours post inoculation (hpi). The soybean cultivar variety Jidou 7 is resistant and susceptible to SMV strain N3 and SC8, respectively. Each experiment was performed with three biological replicates. Each biological replicate contains three plants. The data is represented as mean ± SD (*n*=3). Significant differences were indicated by different lowercase letters, as determined by the LSD test at *p* < 0.05.


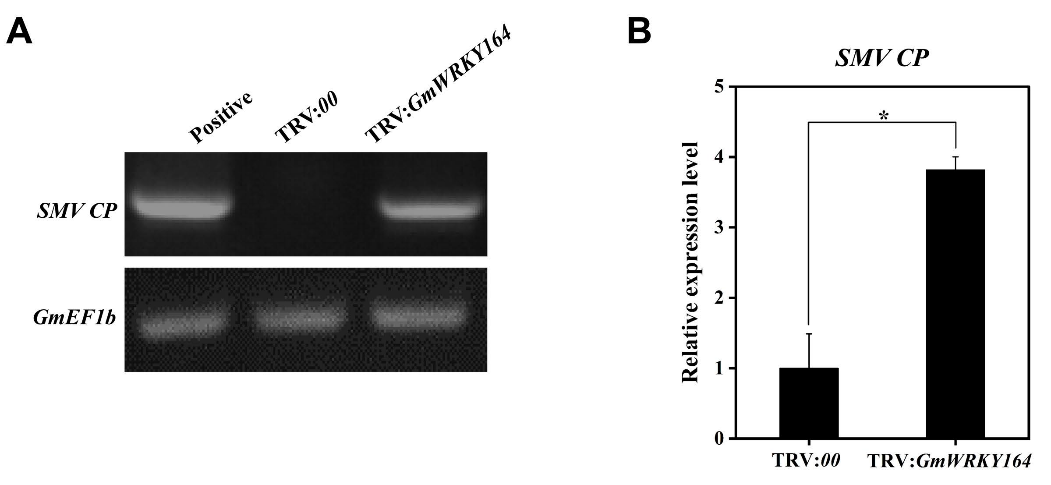


**Figure S2.** Silencing of *GmWRKY164* enhances virus spreading during SMV infection. RT-PCR (A) and RT-qPCR (B) assays of the *CP* gene in the non-inoculated upper leaves. In RT-qPCR assay, the relative expression level of *CP* in TRV:*00* was defined as 1. The soybean cultivar variety Jidou 7 inoculated with N3 was used to detect the expression levels of the *CP* gene. *GmEF1b* was used as a control. Positive represents the leaves inoculated with N3 on Nannong 1138-2 plants. Each experiment was performed with three biological replicates. Each biological replicate contains three plants. The data shown are the means ± SDs (*n*=3). Asterisks (*) indicate a significant difference at *p* < 0.05. The full-length blots are presented in Figure S3 and S4.


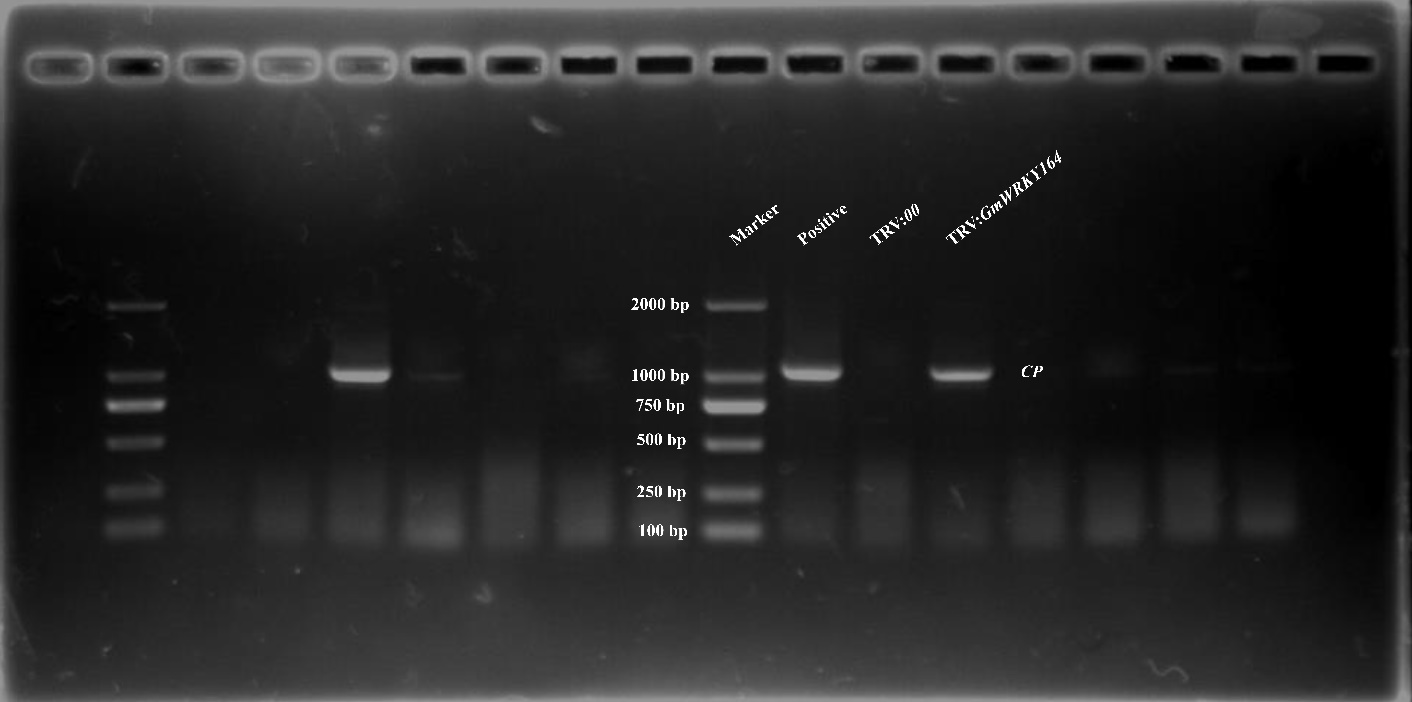


**Figure S3.** Amplification of *CP* gene by RT-PCR separation by agarose gel electrophoresis. The expression levels of the *CP* gene were quantified in the leaves inoculated with N3 on Jidou 7 plants. Positive represents the leaves inoculated with N3 on Nannong 1138-2 plants. The full-length gel of RT-PCR in Figure S2A.


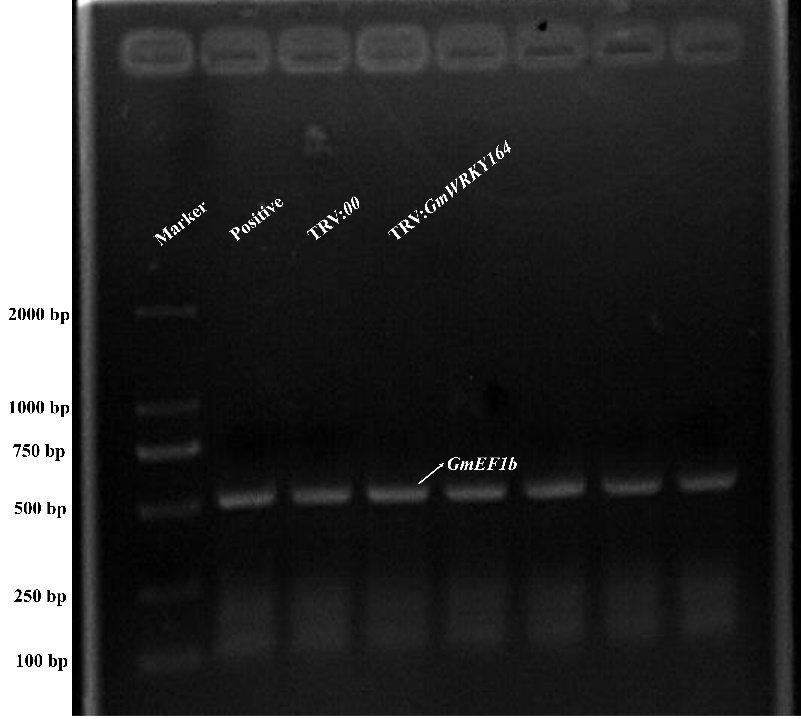


**Figure S4.** Amplification of *GmEF1b* by RT-PCR. The expression levels of the *GmEF1b* gene were quantified in the leaves inoculated with N3 on Jidou 7 plants. Positive represents the leaves inoculated with N3 on Nannong 1138-2 plants. The full-length gel of RT-PCR in Figure S2A.


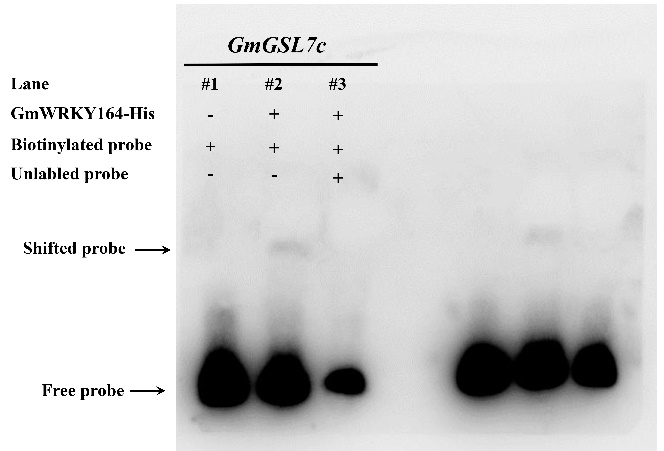


**Figure S5.** The full-length blots of EMSA in Figure 7B. EMSA showing that GmWRKY164 binds directly to the W-box (GTCAA) on the *GmGSL7c* promoter. The W-box element was labeled with biotin and used as a probe. Non-labeled probes (200-fold) were used as competitors. Shifted and free probes indicate the complexes of protein-probes and unbound probes, respectively.


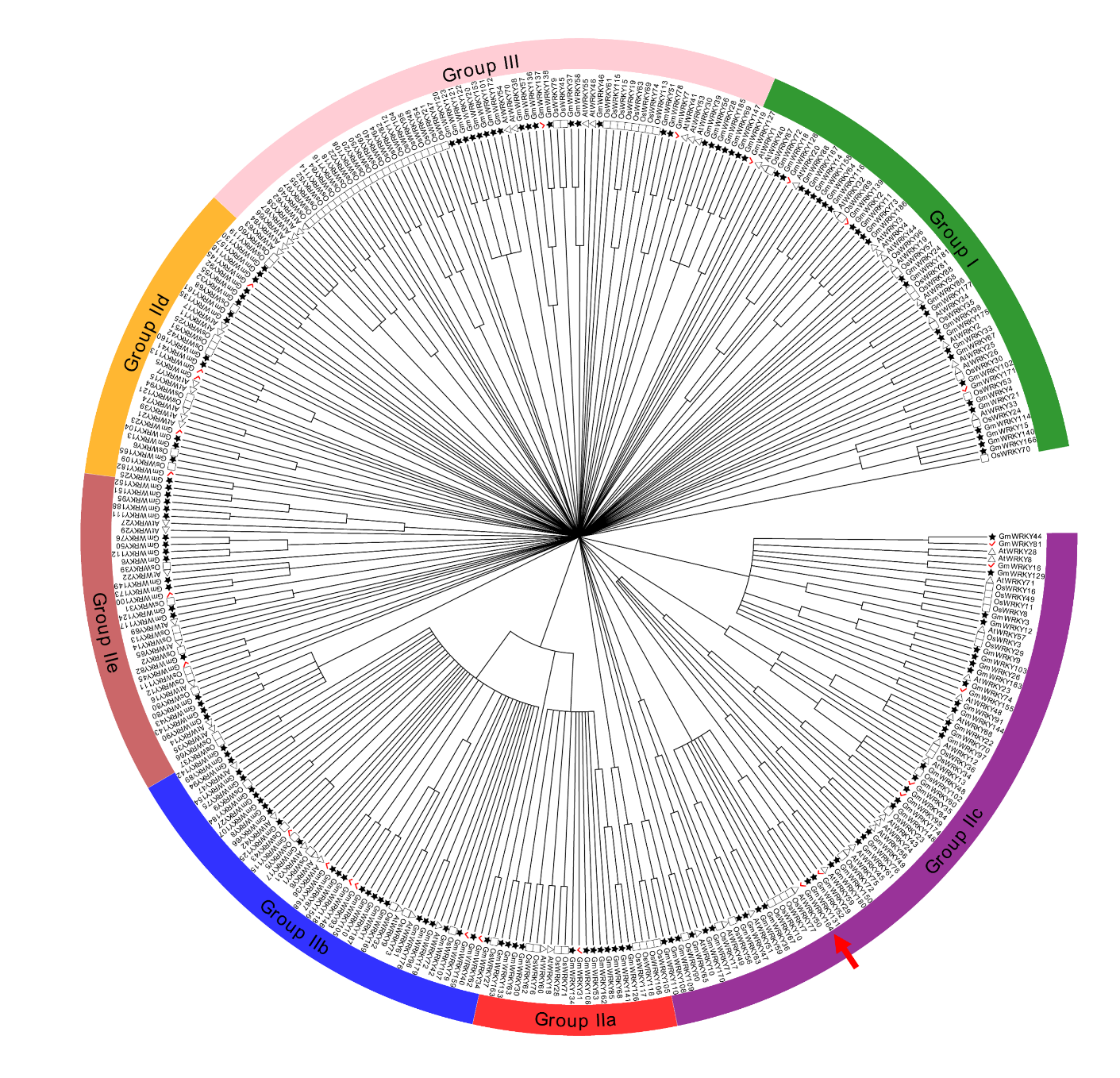


**Figure S6.** Phylogenetic analysis of WRKY proteins on orthologous members from soybean, rice, and *Arabidopsis*. The phylogenetic tree was constructed using the neighbor-joining method with 1000 bootstrap values. The black stars represent WRKYs in soybean, the white triangles represent WRKYs in *Arabidopsis*, and the red check marks represent the 60 *GmWRKYs*, that exhibited significantly differential expression levels in response to SMV infection based on the RNA-seq data. The red arrow points out GmWRKY164 in the phylogenetic tree.
